# Development and Optimization of Polymeric Self-Emulsifying Nanocapsules for Localized Drug Delivery: Design of Experiment Approach

**DOI:** 10.1155/2014/516069

**Published:** 2014-11-24

**Authors:** Jyoti Wadhwa, Abhay Asthana, Sumeet Gupta, Gyati Shilkari Asthana, Ranjit Singh

**Affiliations:** ^1^MM College of Pharmacy, MM University, Mullana, Ambala 133207, India; ^2^Government College of Pharmacy, Rohru, Shimla 171207, India

## Abstract

The purpose of the present study was to formulate polymeric self-emulsifying curcumin nanocapsules with high encapsulation efficiency, good emulsification ability, and optimal globule size for localized targeting in the colon. Formulations were prepared using modified quasiemulsion solvent diffusion method. Concentration of formulation variables, namely, *X*_1_ (oil), *X*_2_ (polymeric emulsifier), and *X*_3_ (adsorbent), was optimized by design of experiments using Box-Behnken design, for its impact on mean globule size (*Y*_1_) and encapsulation efficiency (*Y*_2_) of the formulation. Polymeric nanocapsules with an average diameter of 100–180 nm and an encapsulation efficiency of 64.85 ± 0.12% were obtained. *In vitro* studies revealed that formulations released the drug after 5 h lag time corresponding to the time to reach the colonic region. Pronounced localized action was inferred from the plasma concentration profile (*C*_max_ 200 ng/mL) that depicts limited systemic absorption. Roentgenography study confirms the localized presence of carrier (0–2 h in upper GIT; 2–4 h in small intestine; and 4–24 h in the lower intestine). Optimized formulation showed significantly higher cytotoxicity (IC_50_ value 20.32 *μ*M) in HT 29 colonic cancer cell line. The present study demonstrates systematic development of polymeric self-emulsifying nanocapsule formulation of curcumin for localized targeting in colon.

## 1. Introduction

Accumulated evidences over the years have shown that many anticancer drug molecules, such as steroids and nonsteroidal anti-inflammatory drugs, are associated with numerous side effects. Perhaps the best instance is the cardiovascular impediments imposed by the use of COX inhibitors [1, 2]. As a result, there has been an increasing demand for safer and proficient drug molecules. Curcumin, the Indian solid gold, has been reported as one of the most promising candidates of natural origin, having anticancer properties, with almost no reported side effects [3]. Therapeutic applications of curcumin (CUR), however, remain limited due to its poor absorption and rapid elimination [4]. It has been observed that more than 60–70% CUR is excreted unchanged in the faeces and the remainder is metabolized and absorbed through the intestinal mucosa and liver [5]. Numerous previously reported studies have aimed at improving poor aqueous solubility, bioavailability, alkaline stability, and/or rapid intestinal metabolism of CUR. These includes CUR-impregnated soluble dietary fibres dispersion with enhanced bioavailability (20 times) comparative free CUR [6]. CUR may also be combined with piperine, which inhibits enzymatic conjugation and allows enhanced absorption of unchanged curcuminoids into portal blood [7]. In addition, various strategies have been undertaken to deliver curcumin to intestine by multiparticulate systems [8], solid lipid nanoparticles [9, 10], and polymeric micelles [11] in cancer therapy with enhanced systemic bioavailability. However, these aforementioned systems have poor localisation efficacy due to rapid drug absorption into the systemic circulation.

A review of literature suggests that the application of carrier technology is not limited to scientific interest in such formulations but underlines the potential and versatility in addressing the problems associated with poorly aqueous soluble drugs for localized delivery [12, 13]. New approaches, such as self-emulsifying drug delivery system, have also found their way in enhancing the solubility of CUR in colonic conditions and have several advantages over the existing ones [14, 15]. Moreover, nanocapsules (polymeric wall enveloping an oil core, confining the drug molecule within the central cavity) deliver the drug to target site in a controlled manner [16]. Novel nanocapsules bearing self-emulsification ability and high loading efficiency can effectively address the gastric resistance problems and suitably target colonic sites [17].

To the best of our knowledge, polymeric self-emulsifying nanocapsules for colon targeting have not been explored for curcumin, till date. Nanosized polymeric formulation transformed to emulsified form when it comes in contact with an alkaline medium (simulating colonic region). As a result, dissolved drug might be released in a controlled manner after initial burst release, due to dissolution of polymeric matrix of the nanoparticulate system leading to local action [18]. Present paper reports development and optimization of curcumin polymeric nanocapsule formulation, using a pH-sensitive polymer (HPMCAS-HF) possessing self-emulsifying ability and localized drug delivery in the colon.

## 2. Materials and Methods

### 2.1. Materials

Curcumin (CUR) was purchased from HiMedia, Mumbai, India. Hydroxy propyl methyl cellulose acetate succinate (HPMCAS-HF) was kindly gifted by Arihant trading Co., Mumbai, India. Capryol 90 (C-90), Lauroglycol FCC (LFCC), and Labrafac were obtained* as gratis* from Gattefosse Pvt. Ltd (Mumbai, India). Edible oils (isopropyl myristate, castor oil, oleic acid, ethyl oleate, corn oil, Captex 200, apricot oil, olive oil, and soybean oil) were purchased from HiMedia, Mumbai, India. Aerosil 200 and polyvinyl alcohol (1,25,000 Mol. Wt.) were purchased from CDH, Mumbai, India. 96-well plates (A-U96) were kindly gifted by the Lipidcure Core NOF Corporation, Japan. Potassium dihydrogen phosphate was purchased from Merck, Mumbai, India. All the other chemicals and reagents used in the study were of analytical grade.

### 2.2. Initial Screening of Excipients

#### 2.2.1. Solubility Studies

Solubility of CUR was determined in oils (C-90, LFCC, Labrafac, isopropyl myristate, castor oil, oleic acid, ethyl oleate, corn oil, Captex 200, apricot oil, olive oil, and soybean oil) using saturated shake flask method as reported by Singh et al., 2010 [19]. Excess CUR was suspended in oil in a screw capped glass vial. The mixture was sonicated for 5 min to ensure uniform mixing and solubilization of CUR. The mixture was shaken at 37°C for 24 h in the shaker water bath (Accumax India Pvt. Ltd., New Delhi, India), set at 100 rpm, and allowed to stand for 48 h to attain equilibrium. After 72 h, mixtures were centrifuged at 3000 rpm for 10 min, followed by filtration through a 0.45 *μ*m membrane filter. Analysis was carried out using a Shimadzu HPLC system (LC-2010C HT, Japan) equipped with a reverse phase Phenomenex C18 column (250 mm × 4.6 mm). Elution was carried out at room temperature (37°C), with a UV-visible detection wavelength of 425 nm. A mixture of acetonitrile : HPLC water (57 : 43% v/v), pH 3.3, was used as mobile phase at a flow rate of 1.0 mL/min.

#### 2.2.2. Experimental Design

Based on the preliminary studies, formulation excipients Capryol 90 (oil), HPMCAS-HF (polymer), and Aerosil 200 (adsorbent) were selected as the independent variables *X*_1_, *X*_2_, and *X*_3_, respectively. Box-Behnken design (BBD) was applied to the optimization procedure using Design Expert (Ver. 8.0.7.1) software. A set of seventeen trial formulations (P1–P17) was prepared by varying the concentration of independent variables at three different levels (−1, 0, and +1) (Table 1). The quadratic model generated by the design has (1) as follows:

(1) Y=b0+b1X1+b2X2+b3X3+b12X1X2+b13X1X3 +b23X2X3+b11X12+b22X22+b33X32.

The above equation comprises the coefficient of the intercept, first-order main effect (*X*_1_, *X*_2_, *X*_3_), interaction terms (*X*_1_ *X*_2_, *X*_1_ *X*_3_, *X*_2_ *X*_3_), and higher order effect (*X*_1_^2^, *X*_2_^2^, *X*_3_^2^), where *Y* is the measured response; response variables selected for the optimization purpose were mean globule size (Z-Avg) (*Y*_1_) and encapsulation efficiency (*Y*_2_).

#### 2.2.3. Preparation of Curcumin Loaded Polymeric Self-Emulsifying Nanocapsules (PSN)

**Figure 1:**
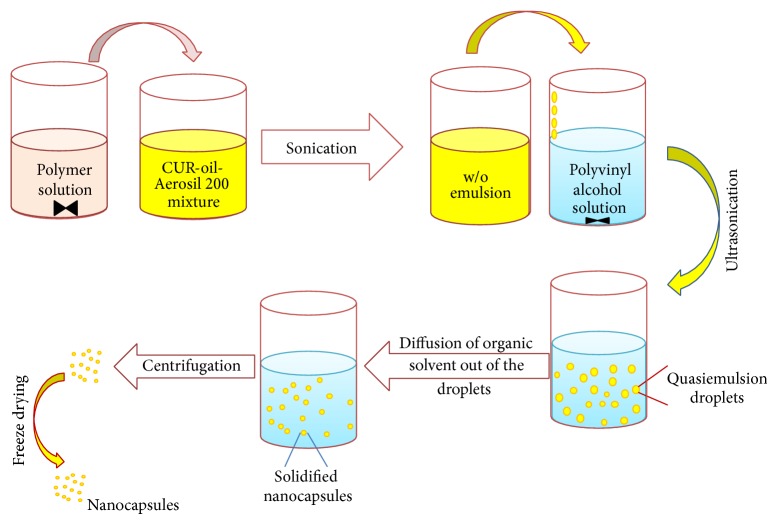
Formulation strategy of CUR-PSN.

CUR loaded PSN formulations (P1–P17) were prepared using modified quasiemulsion solvent diffusion method (Figure 1) [20, 21]. Solution of polymer (HPMCAS-HF) was prepared in ethanol : dichloromethane (1 : 1) mixture. CUR was incorporated in the lipidic phase of Capryol 90, followed by addition of Aerosil 200. CUR-oil mixture was emulsified with HPMCAS-HF solution using probe sonicator to yield a w/o emulsion. An aqueous polyvinyl alcohol solution (0.3% w/v) containing 0.1% sodium lauryl sulfate (SLS) was prepared separately and the prepared w/o emulsion was emulsified in PVA solution, in a dropwise manner. The resulting w/o/w emulsion was stirred magnetically at 500 rpm for 4-5 h at 37°C. Dispersed droplets were solidified by diffusion of the solvent into the aqueous phase. Solidified particles were washed with distilled water thrice followed by centrifugation (25,000 g for 10 minutes at 4°C). Solidified PSN was suspended in distilled water and lyophilized at −18°C for 24 h. Resultant product was stored in a vacuum desiccator at 25°C.

### 2.3. Evaluation of Polymeric Self-Emulsifying Nanocapsules

#### 2.3.1. Globule Size

The globule size of the nanocapsule formulations was determined by photon correlation spectroscopy (PCS), using Zetasizer Nano S90, Malvern, WR14 1XZ, UK. PSN (10 mg) were dispersed in 100 mL of phosphate buffer (pH-7.2) using vortex for 1 h and filtered through a membrane filter (0.22 *μ*m) [15]. The filtrate was analyzed for Z-Avg and zeta potential in triplicate.

#### 2.3.2. Encapsulation Efficiency

CUR loaded PSN formulation theoretically equivalent to 10 mg of CUR was weighed and dissolved in 5 mL of methanol by vortex [22]. Samples were centrifuged (R-4C, Remi centrifuge, Mumbai, India) at 4000 rpm for 10 min, filtered, diluted, and quantified using HPLC. All studies were conducted in triplicate. Encapsulation efficiency was calculated as

(2) Encapsulation  efficiency  % =Actual  drug  loadedTheoretical  drug  loaded∗100.

#### 2.3.3. Emulsification Efficiency

An accurately weighed CUR loaded PSN formulation (equivalent to 10 mg of CUR) was introduced into 100 mL of simulated colonic medium (pH 7.2) [23]. Medium was agitated at 50 rpm for 2 h at 37 ± 0.5°C. Formation of emulsion was observed by visual inspection.

#### 2.3.4. In Process Particulate Distribution

Trinocular phase contrast microscopy was used to determine the distribution of CUR during the process. A droplet of w/o/w emulsion was mounted on a slide and observed by phase contrast microscope (Metzer-M, Mathura, India).

#### 2.3.5. Surface Morphology

The surface morphology of the optimized formulation (P5), with and without plasticizer (TEC), was examined using scanning electron microscope, SEM (EVO 18, Zeiss, Jena, Germany). The sample was fixed using double-sided adhesive tape to a brass specimen and made electrically conductive by gold coating in vacuum [24]. Samples were imaged at different resolutions (2-6 KX).

#### 2.3.6. Differential Scanning Calorimetry

Thermal analysis of CUR, C-90, HPMCAS-HF, physical mixture of CUR with HPMCAS-HF, and optimized PSN formulation (P5) was carried out using differential scanning calorimeter (DSC-204 F1, Phoenix, NETZSCH-Gerätebau GmbH, Deutschland, Germany) under nitrogen purging (50 cc/min). Samples were placed in crimped aluminium pans and were heated from ambient temperature to 250°C at 10°C/min [25].

#### 2.3.7. X-Ray Powder Diffraction (XRPD)

The diffraction pattern of CUR, HPMCAS-HF, physical mixture of CUR with HPMCAS-HF, and optimized formulation (P5) was obtained by XRPD (Bruker D8 Advance, Karlsruhe, Germany) to assess their crystallinity [26]. These were scanned over 2*θ* range from 10 to 35° at 0.05 *θ*/sec step size.

#### 2.3.8. *In Vitro* Dissolution Studies

*In vitro* release of CUR from PSN formulations was determined using USP type II (Paddle type) dissolution apparatus to study the effect of pH on drug release. Formulation P1–P17 equivalent to 10 mg CUR was transferred to 325 mL of dissolution media at 37 ± 0.5°C for 2 h in simulated gastric fluid, pH 1.2; the pH of the dissolution media was then adjusted to 6.8 by the addition of 125 mL of 0.2 M trisodium orthophosphate. Dissolution was continued in phosphate buffer (pH 7.2) up to 12 h. Aliquots of 5 mL of the dissolution medium were withdrawn at predetermined time intervals and filtered through 0.45 *μ*m nylon filter. Concentration of CUR was determined using HPLC as described previously.

In order to discriminate the formulations showing insignificant difference in release profile comparatively optimized formulation (P5), a discriminating study was carried out on formulations (P3, P5, and P10) with aforementioned dissolution conditions, but with varied paddle speed (50 and 75 rpm) and volume of dissolution media (phosphate buffer, pH 7.2), 500 and 900 mL [27].

#### 2.3.9. Cell Viability Assay

The effect of CUR loaded PSN formulation on cell growth was determined on human colon carcinoma, HT29 cell line. The cell growth inhibitory activity of samples was evaluated by 3-(4,5-dimethylthiazol-2-yl)-2,5-diphenyltetrazolium bromide (MTT) colorimetric assay [11]. Cell lines were maintained in folate deficient RPMI 1640 medium under suitable conditions (supplemented with 2 mM glutamine, 1% Pen-Strep (Sigma Aldrich, St. Louis, USA), and 10% fetal bovine serum (FBS)). Cells were maintained at 37°C in a humidified atmosphere containing 5% CO_2_/95% relative humidity (CO_2_ incubator; Binder, Germany). When the cells were confluent, they were trypsinized and seeded into 96-well culture plates at a cell density of 2 × 10^3^ cells per well and the plates were maintained under the conditions previously mentioned. Twenty-four hours later, the old medium was carefully aspirated and the cells were incubated in a logarithmic growth phase with various concentrations ranging from 0 to 30 *μ*g/mL of free CUR, equivalent CUR loaded PSN formulation (P5), equivalent concentration of plain PSN formulation, and DMSO (control). After 24 h of incubation, the old medium was aspirated and replaced with fresh medium. MTT dye (0.5 mg/mL, 20 *μ*L) was added to each well and the plate was incubated for further 4 h at 37°C allowing viable cells to reduce MTT into purple formazan crystal [28]. After incubation, the medium was removed and 200 *μ*L of dimethyl sulphoxide (DMSO) was added and the optical density was measured at 450 nm using a microplate reader (Sunrise Tecan, Männedorf, Switzerland). Cell viability was expressed as a percentage compared to a control that had not been treated with either formulation or free CUR, using the following equation:

(3) $$\begin{array}{c} \%\;\text{Cell}\;\text{Viability}=\frac{N_{i}}{N_{c}}\times 100, \end{array}$$

where *N*_*i*_ and *N*_*c*_ are the numbers of surviving cells in the group treated with CUR loaded formulation and in the untreated cell group, respectively.

#### 2.3.10. *In Vivo* Animal Study

*In vivo* study of free CUR/CUR-loaded PSN formulation was carried out on Duncan Hartley guinea pigs (250–300 g), as per the institutional protocol (MMCP/IAEC/11/23) approved by the animal ethics committee of the MM College of Pharmacy. 100 mg/kg of CUR and equivalent dose of optimized formulation (P5) were administered to guinea pig, in groups of six animals, respectively, in fasting conditions. During the course of the studies, water was available* ad libitum*. Animals used for* in vivo* experiments were divided into three groups (*n* = 6). The PSN suspension (dose 100 mg/kg), suspension of pure CUR (dose 100 mg/kg), and control were administered by oral route using oral feeding needle number 18. Guinea pigs were anesthetized using chloroform, and blood samples (200 *μ*L) were withdrawn from the femoral vein in EDTA coated Eppendorf tubes at specified time intervals (0, 0.5, 1, 2, 4, 6, 8, 10, 12, and 24 h). Blood samples were immediately centrifuged for 5 min and plasma was separated. A Shimadzu LC-2010C HT HPLC system equipped with a UV-visible detector was used to quantitatively determine the concentration of CUR and 4-hydroxybenzophenone (internal standard, IS) in plasma samples. 100 *μ*L of plasma was mixed with 50 *μ*L of IS working solution (8 *μ*g/mL) in an Eppendorf tube. The plasma was then extracted twice with 250 *μ*L of acetonitrile by vigorous mixing for 5 min and was centrifuged at 3000 g for 15 min. 20 *μ*L of the supernatant was injected directly into the column and was analysed quantitatively. Elution was carried out at 300 and 425 nm for 4-hydroxybenzophenone and CUR, respectively [29].

## 3. Results

**Figure 2:**
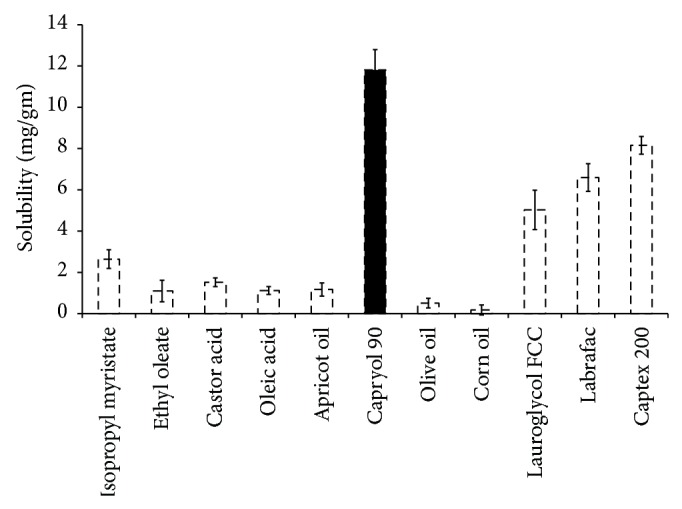
Solubility profile of curcumin in various oils (*n* = 3).

CUR showed a limited aqueous solubility (0.6 *μ*g/mL), whereas it had significantly higher solubility in oils (Figure 2). CUR showed highest solubility (11.41 ± 0.23 mg/g) in C-90 and thereby selected as the oil phase (independent variable *X*_1_) for PSN formulation. On the basis of solubility of polymer in organic solvents (ethanol, acetone, isopropyl alcohol, and dichloromethane), ethanol and dichloromethane (1 : 1) were selected as the organic phase.

### 3.1. Optimization of Formulation Using Design of Experiments

Box-Behnken design (BBD) of experiments was applied to the present study to investigate the effect of independent variables *X*_1_, *X*_2_, and *X*_3_ (oil, polymer, and adsorbent) concentration, respectively, on dependent variables *Y*_1_ and *Y*_2_ (globule size and encapsulation efficiency, resp.) [30]. Analysis of variance (ANOVA) was applied to determine the significance of regression, correlation coefficient (*R*-Square), adjusted *R*-square value, and the adequate precision to estimate *Y*_1_ and *Y*_2_ (Table 2).

### 3.2. Evaluation of Curcumin PSN

#### 3.2.1. Mean Globule Size

Regression analysis for response *Y*_1_ (mean globule size) suggested linear and quadratic model and the cubic model was aliased due to insufficient design points to estimate the coefficients (Table 3). ANOVA data suggested regression to be significant (*P* < 0.01).

The model proposes (4) for globule size

(4) Y1=176.31+12.95X1−8.39X2+8.01X3Y1 −9.343X12+3.34X22−8.20X32+2.64X1X2Y1 −13.49X1X3−6.65X2X3.

(*F* value = 6.531, *R* ^2^ = 0.8935, and adequate precision = 11.009.)

**Figure 3:**
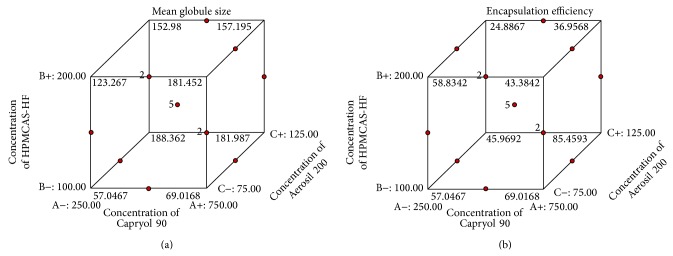
Cube surface graphs for the responses of Capryol 90, HPMCAS-HF, and Aerosil 200. (a) Mean globule size; (b) % encapsulation efficiency.

Globule size was highest in batch P2 at higher levels of oil, low level of polymer, and mid level of adsorbent and was lowest in batch P5 at low levels of oil and adsorbent and mid level of polymer (Figure 3(a)). Response *Y*_1_ was significantly influenced by *X*_1_, *X*_2_, *X*_3_, and *X*_1_ *X*_3_ (Supplementary Table 1 available online at http://dx.doi.org/10.1155/2014/516069).

#### 3.2.2. Encapsulation Efficiency

Regression analysis for response *Y*_2_ (encapsulation efficiency) suggested quadratic model and the cubic model was aliased due to insufficient design points to estimate the coefficients (Table 3). ANOVA data suggested regression to be significant (*P* < 0.01).

The model proposes polynomial equation (5) for percentage drug encapsulation as follows:

(5) Y2=71.31+6.01X1−11.67X2+4.37X3+5.71X12Y2 −6.91X22−17.44X32−6.85X1X2+6.88X1X3Y2 −5.71X2X3.

(*F* value = 10.38, *R* ^2^ = 0.9303, and adequate precision = 14.47.)

Synergistic effects of *X*_1_, *X*_1_ *X*_3_, and *X*_1_^2^ and antagonistic effects of *X*_2_, *X*_3_, *X*_1_ *X*_2_, *X*_2_ *X*_3_, *X*_2_, and *X*_3_^2^ on *Y*_2_ were observed. Encapsulation efficiency was highest in batch P2 at higher levels of oil, low level of polymer, and mid level of adsorbent and was lowest in the batch P12 at mid level of oil and high level of polymer and adsorbent (Figure 3(b)). Significance of results has been confirmed by observed *P* value (<0.03) for each factor (Supplementary Table 1).

#### 3.2.3. Identification and Evaluation of Optimum Formulation Using the Desirability Function

**Figure 4:**
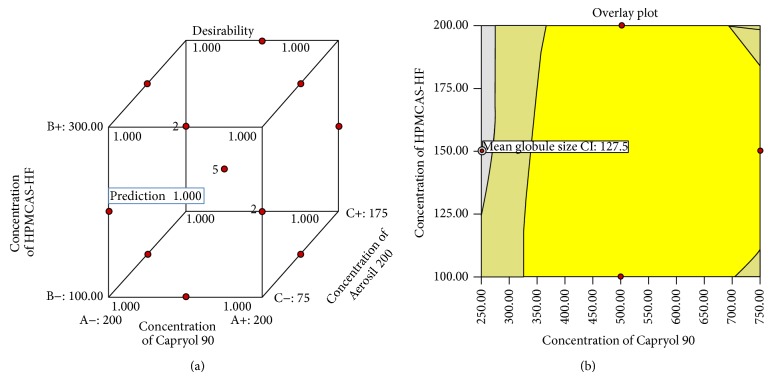
(a) Cube surface graphs for overall desirability (*D*); (b) overlay plot for the optimization of concentration of Capryol 90 and HPMCAS-HF.

PSN formulation with a composition consisting of 250 mg C-90 (oil), 150 mg HPMCAS-HF (polymer), and 75 mg A-200 (adsorbent) was observed to be optimal, in terms of desired mean globule size and encapsulation efficiency (Figure 4(a)).  Figure 4(b) shows the highest desirability factor 1.00 and the overlay plots in a varied range of oil and polymer for optimized formulation.

#### 3.2.4. In Process Particulate Distribution

**Figure 5:**
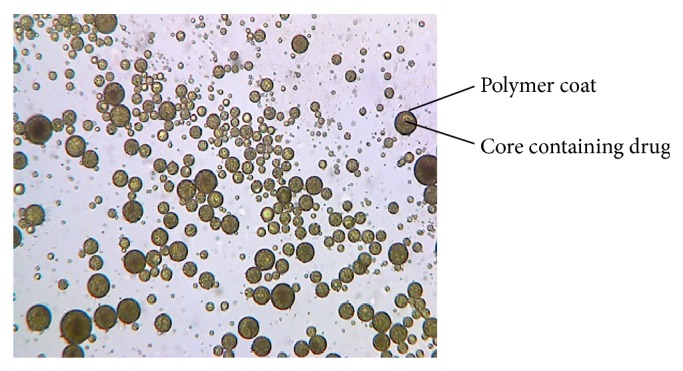
Phase contrast microscopy representing in the process particulate distribution of the emulsion droplets (scale 100x).

Figure 5 shows that the drug particles were uniformly distributed within the nanocapsule. The shell of the particles appears as dark, while the inner core appears as yellowish green.

#### 3.2.5. *In Vitro* Dissolution Studies

**Figure 6:**
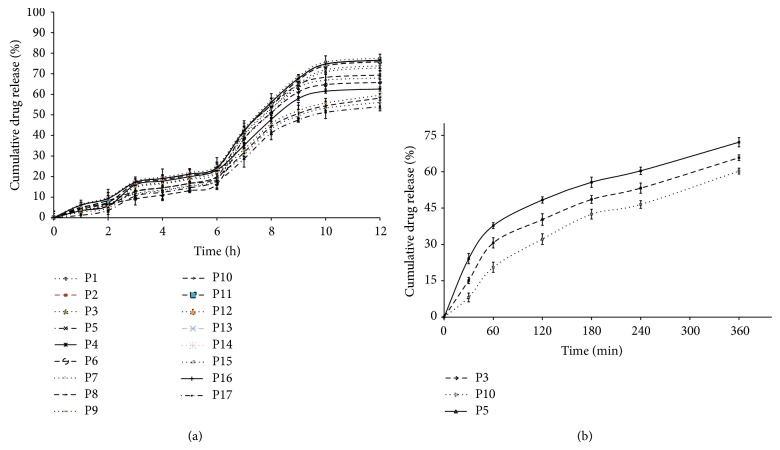
(a) Plot between mean percent curcumin released for all 17 formulations prepared as per 3-factor Box-Behnken design (*n* = 3). (b) Discriminatory dissolution study on PSN formulations (P3, P5, and P10) in phosphate buffer pH 7.2, 900 mL (*n* = 3).

Figure 6(a) illustrates the drug release profile of CUR from PSN formulations (P1–P17). Arround 6% of drug release was observed in 0.1 N HCl (pH 1.2), whereas less than 20% of drug was released in simulated intestinal fluid (pH 6.8). However, insignificant discrimination was observed in selected dissolution test conditions (simulated colonic fluid (pH 7.2).

By increasing the dissolution medium volume (phosphate buffer, pH 7.2) up to 900 mL, the percentage of drug release observed is more than 60% when compared to 500 mL of the same medium at both paddle speeds (50 and 75 rpm). Dissolution profiles of formulations (P3, P5, and P10) in 900 mL of phosphate buffer (pH 7.2) and 50 rpm paddle speed are shown in Figure 6(b). Percentage drug release of three formulations (P5, P3, and P10) in simulated phosphate buffer (pH 7.2; 900 mL) was observed at 72.19 ± 2.13, 65.8 ± 1.58, and 60.27 ± 1.72%, respectively, whereas 58.15 ± 1.86, 56.07 ± 1.12, and 53.99 ± 0.92% of drug release were observed in simulated phosphate buffer (pH 7.2; 500 mL) from three formulations (P5, P3, and P10) respectively.

#### 3.2.6. Differential Scanning Calorimetry (DSC)

**Figure 7:**
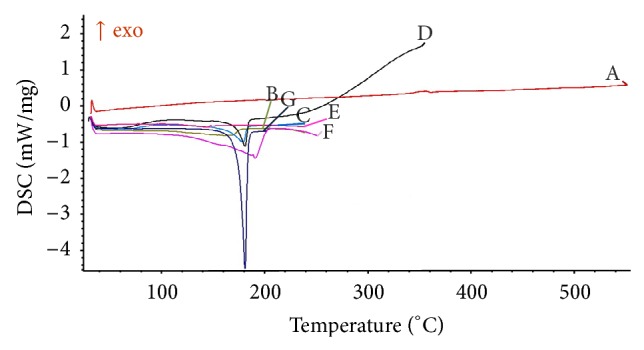
DSC scans of A: Aerosil 200; B: optimized formulation, P5; C: physical mixture (CUR + HPMCAS-HF); D: physical mixture (CUR + HPMCAS-HF + A-200); E: physical mixture (CUR + C-90); F: curcumin; and G: HPMCAS-HF.

DSC thermograms of CUR, HPMCAS-HF, C-90, physical mixture of CUR with HPMCAS-HF, and the optimized formulation (P5) are shown in Figure 7. A sharp endothermic melting peak of CUR appeared at 180.71°C indicating its crystalline nature. HPMCAS-HF and C-90 did not show any peak over the entire range of temperature. Optimized formulation (P5) did not show any melting endothermic peak owing to its amorphous nature.

#### 3.2.7. X-Ray Powder Diffraction (XRPD)

**Figure 8:**
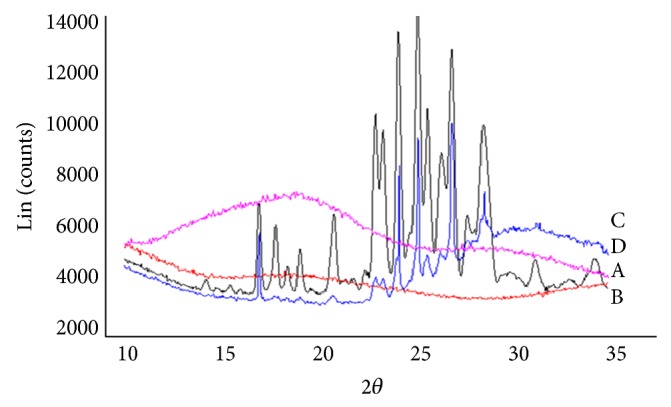
Overlay of XRPD pattern of A. free CUR; B. HPMCAS-HF; C. physical mixture; and D. optimized formulation (P5).

XRPD of CUR describes its crystalline nature. Majority of peaks for CUR occurred at 16.97, 25.20, 16.92, 24.19, 27.02, 25.15, 24.24, 26.97, 17.02, 27.07, 25.30, 23.03, 24.14, 26.92, 22.98, and 28.59° 2*θ*, whereas peak with highest intensity was observed at 25.25 (Figure 8).

#### 3.2.8. Scanning Electron Microscopy (SEM)

**Figure 9:**
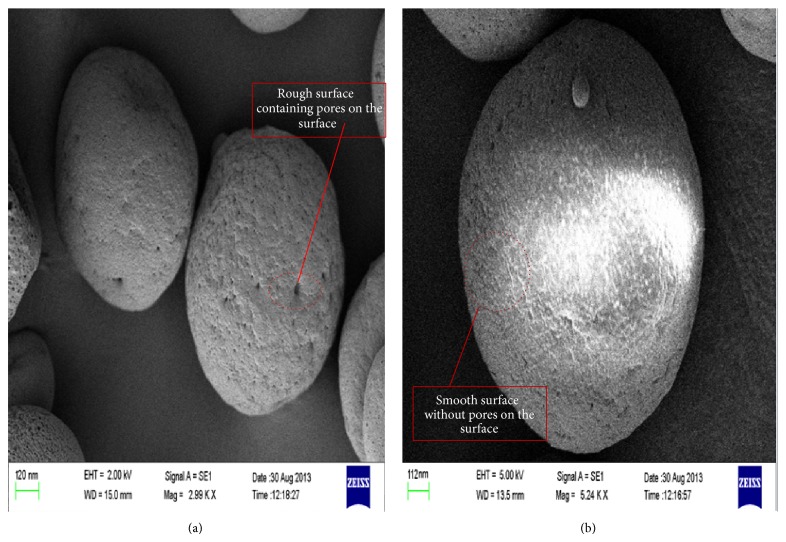
Scanning electron microscopy (SEM) images of optimized formulation (P5) without plasticizer (a) and optimized formulation (P5) with plasticizer (b).

The surface morphology of the optimized formulation (P5) obtained from SEM images is shown in Figure 9. SEM of optimized formulation (P5) with plasticizer appeared with smooth surface, whereas significant pores with rough surface can be seen in images captured for optimized formulation.

#### 3.2.9. Cell Viability Assay

**Figure 10:**
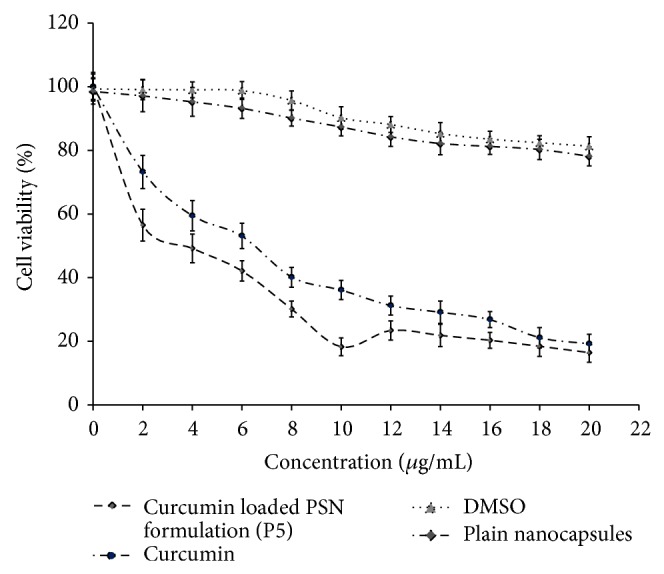
Cell viability study of CUR loaded PSN formulation (P5) on HT29 cell lines (*n* = 3).

Viability of cells was measured using the MTT test to evaluate the cytotoxicity of CUR on HT29 cell lines. Results of cell viability assay are shown in Figure 10. The IC_50_ value of the optimized PSN formulation (P5) was found to be 20.32 micro molar, while that of free CUR had 28.56 micro molar. Compared to DMSO treated cells, no cytotoxicity was observed in the cells exposed to blank PSN formulation. Insignificant change was observed after performing the test in similar manner up to 72 h.

#### 3.2.10. *In Vivo* Animal Study

**Figure 11:**
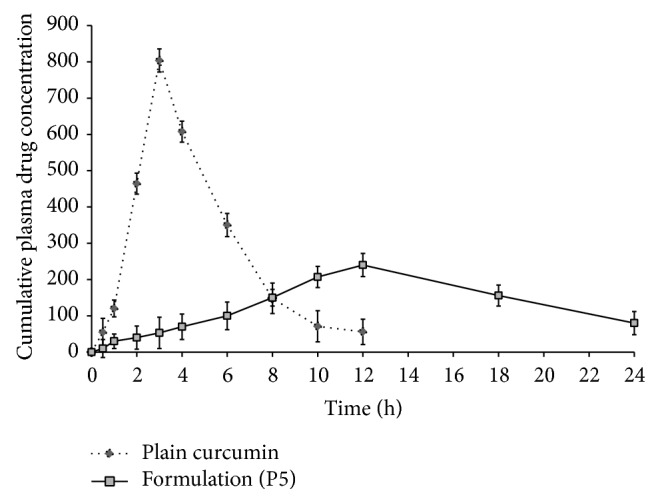
*In vivo* curcumin release from CUR loaded PSN formulation (P5) (*n* = 6).

Plasma concentration time profile of optimized formulation and plain CUR are represented in Figure 11. Pharmacokinetic profile of optimized formulation was evaluated, compared to pure CUR. Concentration of CUR was detectable up to 24 h although insignificant concentration of drug (*C*_max⁡_ 200 ng/mL) was observed which may be due to the slow clearance rate leading to greater enhancement in elimination half-life and correlates well with* in vitro* release data.

**Figure 12:**
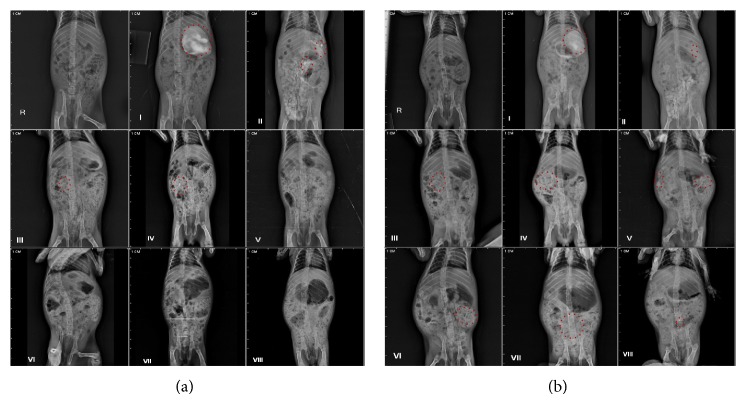
Roentgenography study in guinea pigs. (a) Plain CUR. (b) CUR loaded optimized formulation (PSN) (P5) (where I: blank, II: after 30 min administration of formulation, III: after 1 h administration of formulation, IV: after 2 h administration of formulation, V: after 4 h administration of formulation, VI: after 6 h administration of formulation, VII: after 12 h administration of formulation, and VII: after 24 h administration of formulation).

The results are supported with roentgenographic images (Figure 12) of guinea pig at different time points. Images indicated the presence of contrast which represents the distribution of formulation in GIT at various time points such as from 0 to 2 h in upper GIT, from 2 to 4 h in the small intestine, and from 4 to 24 h in the lower intestinal region (up to anus), whereas plain CUR showed marked availability in lower GIT after 6 h which represents the elimination of the drug.

## 4. Discussion

Polymeric nanocapsules of curcumin were prepared by modified quasiemulsion solvent diffusion method, using an enteric polymer (HPMCAS-HF), which could obviate the issues related to use of high concentration of surfactant (30–70%) in conventional self-emulsifying formulations.

Formation of a fine emulsion is an important parameter to prevent precipitation and recrystallization of the drug from formulation. The rate of emulsification depends on the ratio of polymer and adsorbent and the concentration of oil used in the formulation. The high viscosity of HPMCAS-HF may contribute to the rate of emulsification. Similarly, higher oil concentration increased the interfacial fluidity and accelerates the progress of emulsification [31].

Physiochemical properties, namely, surface morphology, globule size, encapsulation efficiency, and polymorphic change, of nanocapsule formulation should impact drug's release pattern. Release rate is strongly influenced by the surface morphology of drug-loaded nanocapsules. The formulation was observed to exhibit gastric resistance during the first 5 h and thereafter exhibited a controlled release pattern up to 12 h. The controlled release profile might be attributed to smooth surface and delivery mechanism of polymeric nanocapsules probably by surface erosion. It has been observed that higher concentration of polymer extends the duration, due to the formation of crystalline gel at the oil-water interface. This relationship agrees well with the results of Trotta, 1999, who proposed transformation from one liquid crystalline structure to another during the emulsification process [32]. After 5 h lag time, enteric polymer dissolved when the pH was changed to 7.2, corresponding to the pH of the colon. Therefore, considering the GI transit time from stomach to colon of 4 to 6 h, present formulation could serve as potential carriers for delivery of curcumin to colon [33].

The concentration of polymer (HPMCAS-HF) and adsorbent (A-200) was also found to control the rate of drug release. As shown in Figure 6, increasing the amount of polymer as well as the adsorbent resulted in a considerable decrease in drug release. However, insignificant discrimination was observed in selected dissolution test conditions. It may be due to maintenance of nonsink conditions (poor solubility in dissolution media). To establish dissolution test conditions for release characteristics of different formulations, discriminatory study was included. Data observed from discriminatory study showed a significant difference in % cumulative drug release between selected formulations (P3, P5, and P10) with increased volume of dissolution medium (900 mL), whereas the slowest paddle speed (50 rpm) results in steeper drug release profile, typically leading to a higher discriminating efficiency. It may be due to increase in the solubility of drug in enhanced dissolution media (maintenance of sink conditions).

DSC thermograms indicated a change in the physical state of the drug from the crystalline to the amorphous state in PSN formulation. Amorphous state of CUR was further confirmed by the presence of a halo pattern in PXRD, a characteristic of the amorphous form [34]. Appearance of halo pattern (PXRD spectrum) and absence of melting endotherm (DSC thermogram) show that the drug is dispersed in a polymer matrix at a molecular level and stabilized in its amorphous form of polymer [35].

Literature suggests that systems less than 200 *μ*m may be efficiently engulfed by the macrophages present in the colon tissue, thus exhibiting effective localized delivery [36]. Therefore, a polymer formulation with globule size less than 200 *μ*m was chosen for the optimal formulation. Response surface analysis as design of experimental approach was used to identify the effect of variables on globule size and percentage encapsulation efficiency. It has been observed that the mean globule size increased with increase in oil concentration (Capryol 90) and the adsorbent, whereas it decreased with increasing the polymer concentration (HPMCAS-HF). Standardized effects of the Capryol 90 (oil), HPMCAS-HF (polymer), and Aerosil 200 (adsorbent) and their interaction on mean globule size (*Y*_1_) are described in Supplementary Figure 1A.

Drug encapsulation efficiency was found to be affected by the concentration of emulsifier (HPMCAS-HF) as well as the stabilizer (PVA). Formulation with high HPMCAS-HF and PVA concentration showed poor encapsulation efficiency, probably due to an increased formulation viscosity [37]. Furthermore, encapsulation efficiency, increased with increase in oil concentration, however, decreased with increase in concentration of polymer and adsorbent (Supplementary Figure 1B). Design suggested formulation (P5) with all desirable parameters as optimized formulation. Selection was based upon the levels of factors that yielded maximum encapsulation efficiency, optimal globule size, and controlled drug release.

In optimized formulation, localized delivery of CUR was investigated by* in vivo* study in the guinea pig model. Results obtained from plasma drug concentration time profile represent an insignificant amount of drug in plasma (*C*_max⁡_ = 200 ng/mL), suggesting limited systemic uptake of the formulation.

Cytotoxicity assay on drug loaded nanocapsules was conducted on HT29 cell lines to estimate the cell viability with free CUR, blank nanocapsules, and control (DMSO). Results demonstrated that CUR loaded PSN formulation significantly inhibited the growth of the cell lines; however, viability was less than free CUR and DMSO. Blank nanocapsules were treated with cell lines (HT-29) to verify whether a decrease in % viability was due to polymeric nanocapsule or not. It was observed that blank polymeric nanocapsules did not affect the cellular viability of HT-29 cells as compared to PSN formulations. This suggests that PSN formulations release the drug in controlled fashion in the vicinity of proliferating cell lines comparatively blank nanocapsules.

Roentgenographic study was performed on animal model (guinea pig) as this model is analogous to human physiology, also having same transit time (GI to colon) 4–6 h as that of humans. The study has been performed to evaluate the kinetics of PSN formulation in GI. Images indicate that the PSN formulation safely reached colon, which is also presented by formulations,* in vitro*, with sufficient gastric resistance and lag time. The evaluation of the results obtained at different stages (time points) suggests that PSN formulation is present starting from initial time point (i.e., 30 min) till 24th h. It can be observed that the formulation reaches intestinal region after 4 h (Figure 12(b) (V)), whereas, in case of CUR, there is no such indication. Further, Figure 12(b) (VIII) represents PSN formulation in lower intestine up to 24 h.

Results favour the conditions, such as limited systemic absorption (plasma drug profile), intact drug until reaching the large intestine (roentgenographic study), and effective delivery of drug to target site (cell line study), required for localized delivery [36]. Therefore, it can be concluded that the developed formulation could be considered as a promising delivery strategy towards localized targeting of CUR to colonic region for the effective treatment of colorectal pathologies.

## 5. Conclusion

In the present study, a polymeric self-emulsifying nanocapsule formulation of curcumin was successfully developed. Optimization of oil, polymeric emulsifier, and surfactant was undertaken by Box-Behnken design, finally generating an optimal formula for nanocapsule formulation. Capryol 90 (oil phase), 250 mg, HPMCAS-HF (polymeric emulsifier), 250 mg, and Aerosil 200 (adsorbent), 75 mg, were opted for optimized formulation. Optimized formulation follows the conditions required to deliver a drug in the colon locally. Roentgenographic studies are in close agreement with* in vitro* dissolution studies. The present work paved way to coin a methodology for the systematic development of polymeric self-emulsifying nanocapsule for localized delivery in colonic region.

## Supplementary Material

A supplementary table showing the ANOVA results for responses, mean globule size (nm) and encapsulation efficiency of nanocapsules (%) (n = 3). A supplementary figure showing standardized effects of the independent variables on dependent variables.

## Figures and Tables

**Table 1:** Composition of nanocapsule formulation using Box-Behnken design.

Levels	Low		Middle		High	
	Coded	Actual (mg)	Coded	Actual (mg)	Coded	Actual (mg)
Independent factors						
*X*_1_: conc. of Capryol 90 (oil) (mg)	−1	250	0	500	1	750
*X*_2_: conc. of HPMCAS-HF (polymeric emulsifier) (mg)	−1	100	0	150	1	200
*X*_3_: conc. of Aerosil 200 (adsorbent) (mg)	−1	75	0	100	1	125
Dependent variables						
*Y*_1_: mean globule size (nm)			**Minimize**			
*Y*_2_: encapsulation efficiency (%)			**Maximize**			

**Table 2:** Combination levels of independent variables and the outcomes of response variables by Box-Behnken design.

Batch number	Independent factors			Actual value		Predicted value	
	Capryol 90	HPMCAS-HF	Aerosil 200	Mean globule size (nm)	Encapsulation efficiency (%)	Mean globule size (nm)	Encapsulation efficiency (%)
P1	−1	−1	0	173.86	67.42	168.40	68.95
P2	+1	−1	0	194.84	96.18	189.01	94.68
P3	−1	+1	0	140.49	57.81	146.31	59.30
P4	+1	+1	0	172.06	59.15	177.51	57.61
P5	−1	0	−1	127.50	71.82	124.31	64.85
P6	+1	0	−1	180.02	67.05	177.20	63.11
P7	−1	0	+1	164.52	38.41	167.33	42.34
P8	+1	0	+1	163.07	61.16	166.25	68.12
P9	0	−1	−1	156.55	51.86	165.25	57.29
P10	0	+1	−1	164.35	39.90	161.70	45.36
P11	0	−1	+1	191.87	65.44	194.51	59.97
P12	0	+1	+1	173.07	30.61	164.43	25.18
P13	0	0	0	173.07	71.01	176.31	71.31
P14	0	0	0	180.91	70.79	176.31	71.31
P15	0	0	0	169.87	69.76	176.31	71.31
P16	0	0	0	184.81	75.05	176.31	71.31
P17	0	0	0	172.92	69.96	176.31	71.31

^*^Standard deviation of the observed responses was within ±5% (*n* = 3).

**Table 3:** Regression analysis for mean globule size and encapsulation efficiency.

Response	Model	Std. dev.	*R* ^ 2^	Adjusted *R* ^2^	Remarks
Mean globule size (nm)	Linear	12.79	0.532	0.424	Suggested
	Second order	10.93	0.737	0.579	—
	Quadratic	8.31	0.893	0.756	Suggested
	Cube	6.26	0.965	0.862	Aliased

Encapsulation efficiency (%)	Linear	13.60	0.389	0.248	—
	Second order	13.77	0.518	0.229	—
	Quadratic	6.26	0.930	0.840	Suggested
	Cube	2.15	0.995	0.981	Aliased

## Abbreviations

CUR: Curcumin
PSN: Polymeric self-emulsifying nanocapsules
XRPD: X-ray powder diffraction
HPMCAS-HF: Hydroxy propyl methyl cellulose acetate succinate-HF
SGF: Simulated gastric fluid
SIF: Simulated intestinal fluid
FbD: Formulation by design
CAS: Chemical abstract service
LFCC: Lauroglycol FCC
C-90: Capryol 90
RSM: Response surface methodology
BBD: Box-Behnken design
A-200: Aerosil 200
DS: Design space
QbD: Quality by design
HPLC: High performance liquid chromatography
PCS: Photon correlation spectroscopy
SEM: Scanning electron microscope
TEM: Transmission electron microscope
PVA: Polyvinyl alcohol
GIT: Gastrointestinal tract
ICH: International conference on harmonization
COX: Cyclooxygenase.

## References

[1] Moodley I. (2008). Review of the cardiovascular safety of COXIBs compared to NSAIDS. *Cardiovascular Journal of Africa*.

[2] Dogne J. M., Supuran C. T., Pratico D. (2005). Adverse cardiovascular effects of the coxibs. *Journal of Medicinal Chemistry*.

[3] Aggarwal B. B., Harikumar K. B. (2009). Potential therapeutic effects of curcumin, the anti-inflammatory agent, against neurodegenerative, cardiovascular, pulmonary, metabolic, autoimmune and neoplastic diseases. *International Journal of Biochemistry and Cell Biology*.

[4] Aggarwal B. B., Bhatt I. D., Ichikawa H. (2006). Curcumin-biological and medicinal properties. *Turmeric: The Genus Curcuma*.

[5] Rodriguez J. C., Santibanez D., Narayanan S. (2008). Ginger and curcumin in cancer prevention. *Botanical Medicine in Clinical Practice*.

[6] Krishnakumar I. M., Ravi A., Kumar D., Kuttan R., Maliakel B. (2012). An enhanced bioavailable formulation of curcumin using fenugreek-derived soluble dietary fibre. *Journal of Functional Foods*.

[7] Shoba G., Joy D., Joseph T., Majeed M., Rajendran R., Srinivas P. S. S. R. (1998). Influence of piperine on the pharmacokinetics of curcumin in animals and human volunteers. *Planta Medica*.

[8] Chuah L. H., Billa N., Roberts C. J., Burley J. C., Manickam S. (2013). Curcumin-containing chitosan nanoparticles as a potential mucoadhesive delivery system to the colon. *Pharmaceutical Development and Technology*.

[9] Chirio D., Gallarate M., Peira E., Battaglia L., Serpe L., Trotta M. (2011). Formulation of curcumin-loaded solid lipid nanoparticles produced by fatty acids coacervation technique. *Journal of Microencapsulation*.

[10] Guri A., Gülseren I., Corredig M. (2013). Utilization of solid lipid nanoparticles for enhanced delivery of curcumin in cocultures of HT29-MTX and Caco-2 cells. *Food and Function*.

[11] Gou M., Men K., Shi H. (2011). Curcumin-loaded biodegradable polymeric micelles for colon cancer therapy *in vitro* and *in vivo*. *Nanoscale*.

[12] Sylvester A., Sivaraman B., Deb P., Ramamurthi A. (2013). Nanoparticles for localized delivery of hyaluronan oligomers towards regenerative repair of elastic matrix. *Acta Biomaterialia*.

[13] Barrias C. C., Lamghari M., Granja P. L., Sá Miranda M. C., Barbosa M. A. (2005). Biological evaluation of calcium alginate microspheres as a vehicle for the localized delivery of a therapeutic enzyme. *Journal of Biomedical Materials Research*.

[14] Zhang L., Zhu W., Yang C., Guo H., Yu A., Ji J., Gao Y., Sun M., Zhai G. (2012). A novel folate-modifed self-microemulsifying drug delivery system of curcumin for colon targeting. *International Journal of Nanomedicine*.

[15] Huang Y., Tian R., Hu W., Jia Y., Zhang J., Jiang H., Zhang L. (2013). A novel plug-controlled colon-specific pulsatile capsule with tablet of curcumin-loaded SMEDDS. *Carbohydrate Polymers*.

[16] Mora-Huertas C. E., Fessi H., Elaissari A. (2010). Polymer-based nanocapsules for drug delivery. *International Journal of Pharmaceutics*.

[17] Prego C., Fabre M., Torres D., Alonso M. J. (2006). Efficacy and mechanism of action of chitosan nanocapsules for oral peptide delivery. *Pharmaceutical Research*.

[18] Asghar L. F., Chandran S. (2006). Multiparticulate formulation approach to colon specific drug delivery: current perspectives. *Journal of Pharmacy & Pharmaceutical Sciences*.

[19] Singh S. K., Prasad Verma P. R., Razdan B. (2010). Glibenclamide-loaded self-nanoemulsifying drug delivery system: development and characterization. *Drug Development and Industrial Pharmacy*.

[20] Nocent M., Bertocchi L., Espitalier F., Baron M., Couarraze G. (2001). Definition of a solvent system for spherical crystallization of salbutamol sulfate by quasi-emulsion solvent diffusion (QESD) method. *Journal of Pharmaceutical Sciences*.

[21] You J., Cui F. D., Han X., Wang Y. S., Yang L., Yu Y. W., Li Q. P. (2006). Study of the preparation of sustained-release microspheres containing zedoary turmeric oil by the emulsion-solvent-diffusion method and evaluation of the self-emulsification and bioavailability of the oil. *Colloids and Surfaces B: Biointerfaces*.

[22] Youm I., Yang X. Y., Murowchick J. B., Youan B.-B. C. (2011). Encapsulation of docetaxel in oily core polyester nanocapsule intended for breast cancer therapy. *Nanoscale Research Letters*.

[23] Taha E. I., Al-Saidan S., Samy A. M., Khan M. A. (2004). Preparation and in vitro characterization of self-nanoemulsified drug delivery system (SNEDDS) of all-trans-retinol acetate. *International Journal of Pharmaceutics*.

[24] Craig D. Q. M., Barker S. A., Banning D., Booth S. W. (1995). An investigation into the mechanisms of self-emulsification using particle size analysis and low frequency dielectric spectroscopy. *International Journal of Pharmaceutics*.

[25] Barari M., Faridi-Majidi R., Madani M., Sharifi-Sanjani N., Oghabian M. A. (2009). Preparation of nanocapsules via emulsifier-free miniemulsion polymerization. *Journal of Nanoscience and Nanotechnology*.

[26] Zaske L., Perrin M. A., Leveiller F. (2001). Docetaxel: solid state characterization by X-ray powder diffraction and thermogravimetry. *Journal de Physique Archives*.

[27] Lagace M., Gravelle L., Di Maso M., McClintock S. (2004). Developing a discriminating dissolution procedure for a dual active pharmaceutical product with unique solubility characteristics. *Dissolution Technologies*.

[28] Sgouras D., Duncan R. (1990). Methods for the evaluation of biocompatibility of soluble synthetic polymers which have potential for biomedical use: 1—Use of the tetrazolium-based colorimetric assay (MTT) as a preliminary screen for evaluation of *in vitro* cytotoxicity. *Journal of Materials Science: Materials in Medicine*.

[29] Ma Z., Shayeganpour A., Brocks D. R., Lavasanifar A., Samuel J. (2007). High-performance liquid chromatography analysis of curcumin in rat plasma: application to pharmacokinetics of polymeric micellar formulation of curcumin. *Biomedical Chromatography*.

[30] Nazzal S., Khan M. A. (2002). Response surface methodology for the optimization of ubiquinone self-nanoemulsified drug delivery system. *AAPS PharmSciTech*.

[31] James-Smith M. A., Alford K., Shah D. O. (2007). A novel method to quantify the amount of surfactant at the oil/water interface and to determine total interfacial area of emulsions. *Journal of Colloid and Interface Science*.

[32] Trotta M. (1999). Influence of phase transformation on indomethacin release from microemulsions. *Journal of Controlled Release*.

[33] Patel M. M., Amin A. F. (2011). Formulation and development of release modulated colon targeted system of meloxicam for potential application in the prophylaxis of colorectal cancer. *Drug Delivery*.

[34] Hancock B. C., Zografi G. (1994). The relationship between the glass transition temperature and the water content of amorphous pharmaceutical solids. *Pharmaceutical Research*.

[35] Bansal S. S., Kaushal A. M., Bansal A. K. (2008). Co-relationship of physical stability of amorphous dispersions with enthalpy relaxation. *Pharmazie*.

[36] Mrsny R. J., Friend D. R. (1992). Drug absorption in the colon a critical review. *Oral Colon-Specific Drug Delivery*.

[37] Devrim B., Canefe K. (2006). Preparation and evaluation of modified release ibuprofen microspheres with acrylic polymers (Eudragit) by quasiemulsion solvent diffusion method: effect of variables. *Acta Poloniae Pharmaceutica*.

