# Supplementary material for: Development and Optimization of Polymeric Self-Emulsifying Nanocapsules for Localized Drug Delivery: Design of Experiment Approach
Source: ScientificWorldJournal. 2014 Nov 24;2014:516069. doi: 10.1155/2014/516069 (PMC4265377; doi:10.1155/2014/516069)

Supplementary File

Table 1: Summary of each factor effect and its p-values for responses Mean globule size (nm) and Encapsulation Efficiency (%)

**Factor**	**Mean globule size (nm)**		**Encapsulation Efficiency (%)**	
	**Factor effect**	**p value**	**Factor effect**	**p value**
Intercept	176.31		71.31	
X_1[@]	12.95	0.0031	6.01	0.030
X_2[$]	-8.39	0.0245	-11.67	0.0012
X_3[*]	8.01	0.0295	-4.37	0.088
X_1X_2[ε]	2.64	0.5444	-6.85	0.0647
X_1X_3[ε]	-13.49	0.0141	6.88	0.064
X_2X_3[ε]	-6.65	0.1537	-5.71	0.110
X_1^2[α]	-9.343	0.0545	5.74	0.102
X_2^2[α]	3.34	0.4370	-6.91	0.057
X_3^2[α]	-8.195	0.0828	-17.44	0.0007

[@] Capryol 90 (oil); [$] HPMCAS-HF (polymer); *Aerosil 200 (adsorbent)
[ε]Interaction terms; [α]high order effect

Figure 1 [A]: Evaluation of the standardized effects of the Capryol® 90 **(X_1),** HPMCAS-HF **(X_2) and** Aerosil® 200 **(X_3), and their interactions on mean globule size (Y_1)**

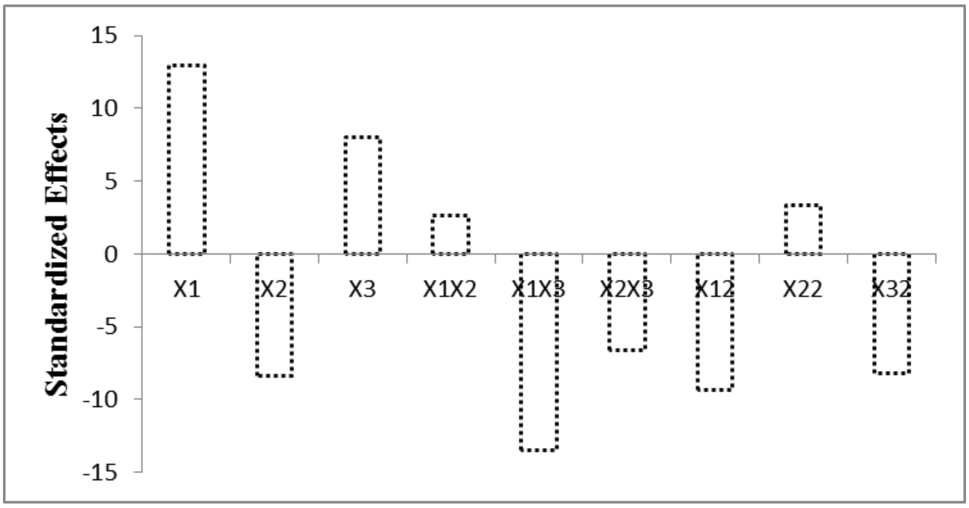

Figure 1 [B]: Evaluation of the standardized effects of the Capryol® 90 **(X_1),** HPMCAS-HF **(X_2) and** Aerosil® 200 **(X_3), and their interactions on encapsulation efficiency (Y_2)**

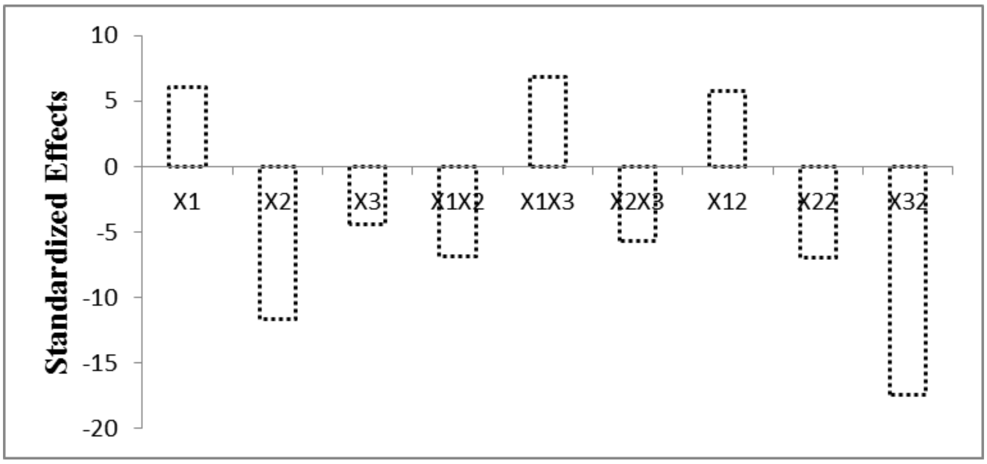

Supplement: Supplementary file 1 — A supplementary table showing the ANOVA results for responses, mean globule size (nm) and encapsulation efficiency of nanocapsules (%) (n = 3). A supplementary figure showing standardized effects of the independent variables on dependent variables. [file 516069.f1.pdf]
